# Infected left ventricular thrombus confirmed by FDG PET/CT presenting as persistent *Streptococcus anginosus* bacteraemia in ischaemic cardiomyopathy: a case report

**DOI:** 10.1093/ehjcr/ytaf579

**Published:** 2025-11-06

**Authors:** Nithin George

**Affiliations:** Department of Cardiology, Lyell McEwin Hospital, Haydown Road, Elizabeth Vale, Adelaide, SA 5112, Australia

**Keywords:** Case report, Infected thrombus, Heart failure, Ischaemic cardiomyopathy, Echocardiography, FDG PET /CT, *Streptococcus anginosus*, Anticoagulation, Antibiotics, Sepsis

## Abstract

**Background:**

Infected left ventricular (LV) thrombus is a rare but serious complication in patients with heart failure with reduced ejection fraction (HFrEF). It is associated with persistent bacteraemia, systemic embolization, and stroke. Diagnosis requires a high index of clinical suspicion, supported by multimodal imaging and coordinated multidisciplinary management.

**Case Summary:**

We report a 69-year-old man with ischaemic cardiomyopathy (EF 30%), prior multi-territory stroke due to LV thrombus, and multiple comorbidities, who was admitted with reduced GCS following ICU treatment for *Streptococcus anginosus* septic shock. Despite several antibiotic courses, he experienced recurrent bacteraemia over 6 months with no clear source. Given his history, an infected LV thrombus and infective endocarditis were considered. Transthoracic echocardiography (TTE) demonstrated recurrence of an apical thrombus, which was confirmed on transoesophageal echocardiography (TOE) without valvular vegetations. FDG positron emission tomography (PET) showed focal uptake at the LV apex in a circular pattern with central photopenia, consistent with an infected thrombus. He was managed with 6 weeks of intravenous benzylpenicillin followed by 6 months of oral amoxicillin, alongside warfarin anticoagulation. Surgery was not pursued due to frailty and comorbidities. He improved clinically with the resolution of bacteraemia. Follow-up PET and TTE were planned to assess the response.

**Discussion:**

This case represents one of the few PET-confirmed infected LV thrombus presenting with persistent *S. anginosus* bacteraemia. It highlights the diagnostic value of multimodal imaging, particularly PET/CT in distinguishing infected thrombus from sterile thrombus and endocarditis, and underscores the importance of prolonged antibiotic therapy, anticoagulation, and multidisciplinary management.

Learning points

- Infected left ventricular thrombus is a rare but clinically significant source of persistent bacteraemia, particularly in patients with ischaemic cardiomyopathy and prior thromboembolic events.
- Recurrent *Streptococcus anginosus* bacteraemia with no obvious source should prompt evaluation for deep-seated cardiac infection, including infected thrombus.
- Multimodal imaging, particularly FDG-PET/CT, plays a pivotal role in confirming thrombus infection when echocardiographic findings are inconclusive.
- Management requires prolonged pathogen-directed antibiotic therapy in combination with therapeutic anticoagulation, with close follow-up to monitor for thrombus resolution.
- A multidisciplinary approach involving cardiology, infectious diseases, and imaging specialists is essential for optimal diagnostic and therapeutic outcomes.

## Introduction

Infected left ventricular (LV) thrombus is a rare but clinically significant complication among patients with heart failure with reduced ejection fraction (HFrEF). Such cases are associated with persistent infection, recurrent systemic embolism, and significant morbidity.^1,2^ Clinical suspicion, multimodal imaging, and timely therapeutic intervention are critical for successful diagnosis and management.^3^ Here, we present a challenging case illustrating the diagnostic complexity, emphasizing the pivotal role of imaging and multidisciplinary management.

## Summary figure

**Figure:**
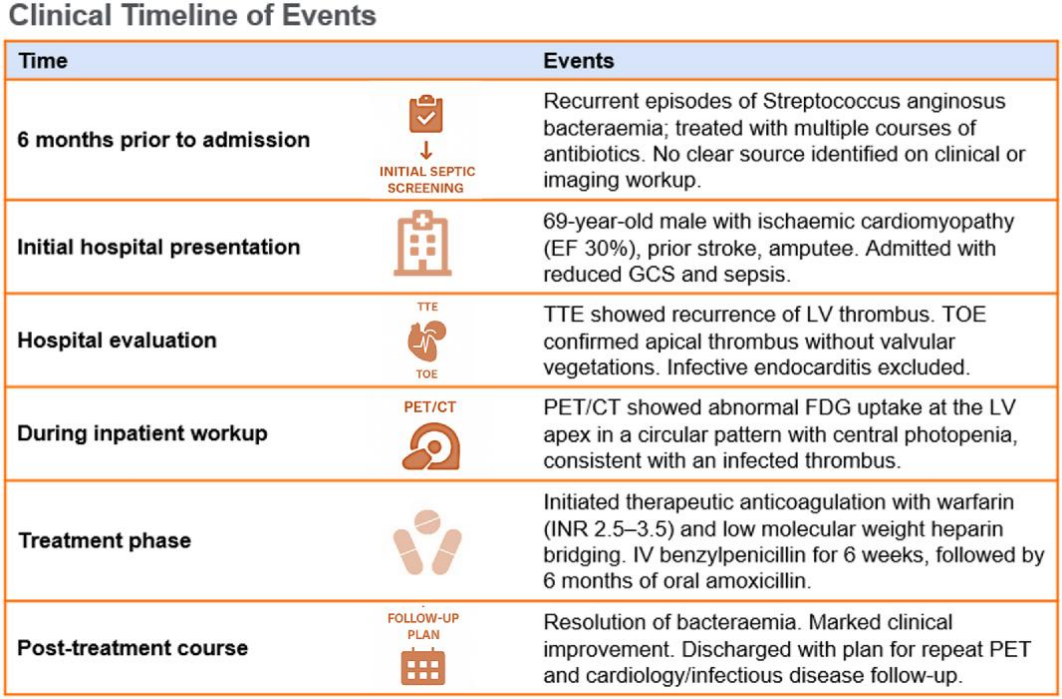


## Case presentation

A 69-year-old man from a nursing home presented to our institution with reduced Glasgow Coma Scale following a recent ICU admission for septic shock complicated by delirium. His medical background included severe ischaemic cardiomyopathy (EF 30%) managed medically, prior multi-territory stroke attributed to LV thrombus, triple-vessel coronary disease, hypertension, type 2 diabetes, and left upper-limb amputation, resulting in significant baseline functional impairment.

Over ∼6 months, he had experienced multiple hospital admissions for *Streptococcus anginosus* bacteraemia at roughly 2 month intervals, with recurrent positive blood cultures despite prolonged intravenous and oral antibiotics. Infectious diseases teams performed comprehensive investigations including dental review, abdominal ultrasound, urinary tract imaging, and CT chest-abdomen-pelvis which did not identify a clear infective focus. Given his prior thrombus-related stroke, there was clinical suspicion of infective endocarditis or an infected thrombus.

**Figure 1:**
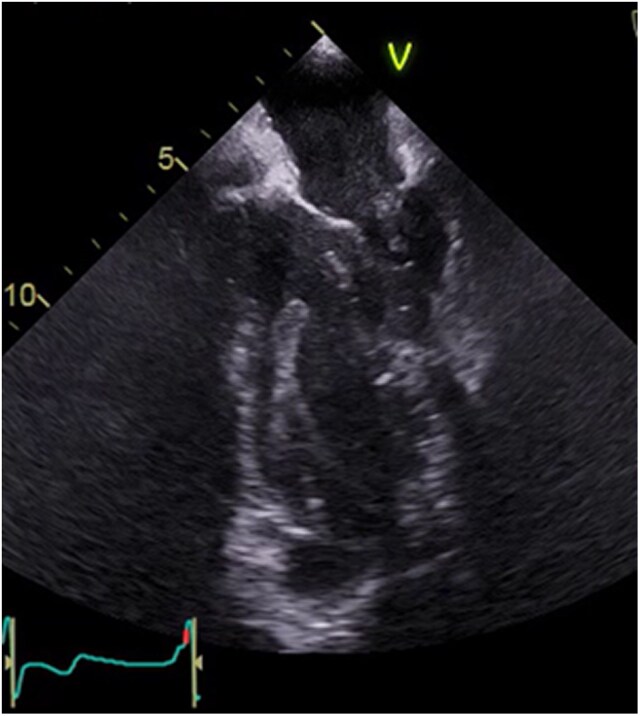
Mid-oesophageal two-chamber TOE view showing a mobile left ventricular thrombus tethered to the apical cap.

**Figure 2:**
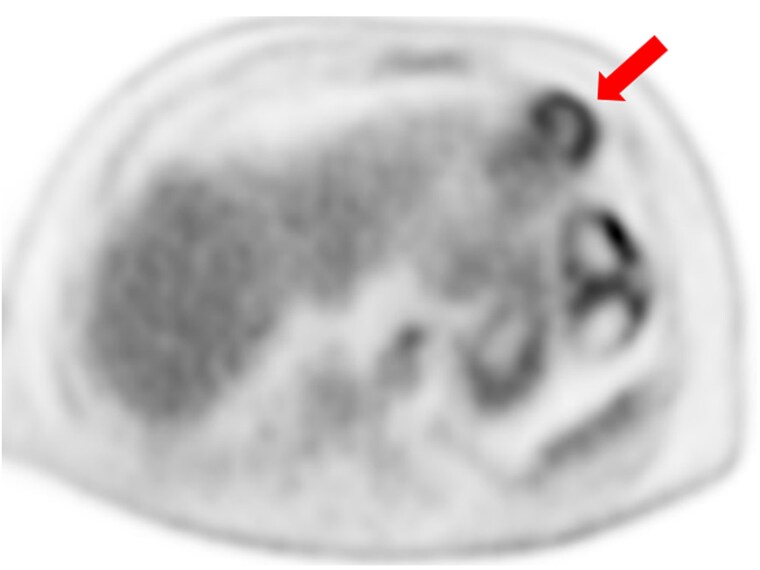
Axial PET image showing focal FDG uptake at the left ventricular apex with a central photopenic core, consistent with an avascular thrombus centre.

**Figure 3:**
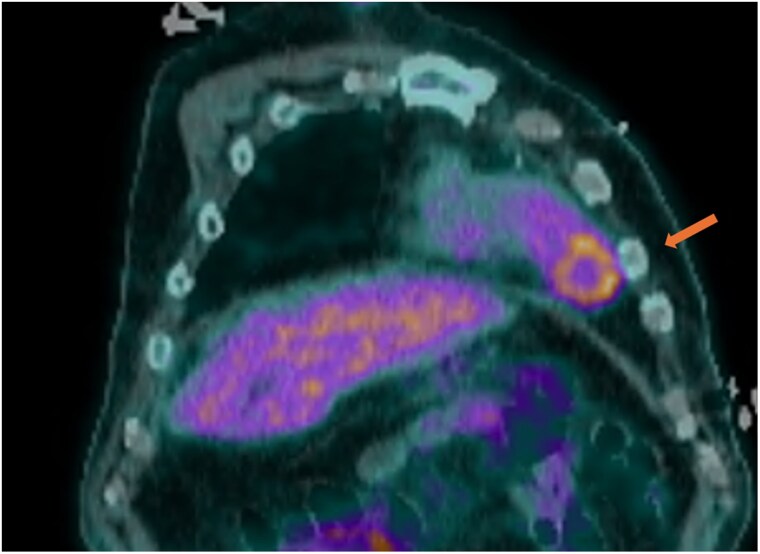
Fused coronal PET/CT image demonstrating a circular FDG-avid rim at the apex, representing peripheral inflammatory activity around an infected thrombus.

**Figure 4:**
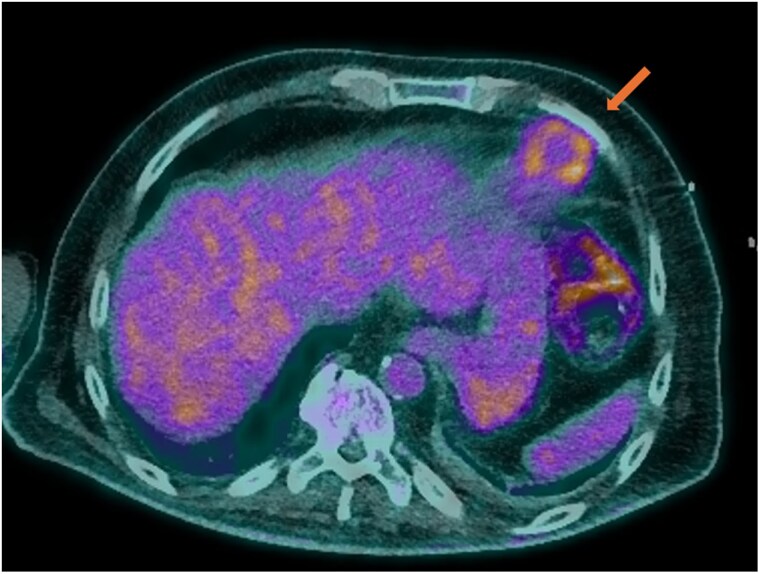
Fused axial PET/CT image demonstrating a circular FDG-avid rim in LV apex.

Repeat transthoracic echocardiography (TTE) demonstrated recurrence of LV thrombus, previously documented as resolved with anticoagulation therapy. Subsequent transoesophageal echocardiography (TOE) confirmed a thrombus without evidence of valvular vegetations (*Figure 1*). Positron emission tomography (PET) using 18F-FDG (fluorodeoxyglucose) imaging demonstrated increased FDG uptake at the LV apex, confirming infection of the thrombus (*Figures 2–4*), with no abnormal uptake elsewhere.

Management involved prolonged intravenous benzylpenicillin (1.8 g every 4 h) therapy for six weeks, followed by oral amoxicillin (1 g three times daily) for an additional 6 months based on the *S. anginosus* susceptibility profile. Warfarin anticoagulation was maintained targeting an INR of 2.5–3.5, with bridging using low-molecular-weight heparin when indicated. Surgical intervention was not pursued, as the patient was deemed a poor operative candidate due to frailty and comorbidities. The patient’s clinical condition improved with the resolution of recurrent bacteraemia. Further PET imaging was planned at 6 months post-treatment to confirm thrombus resolution.

## Discussion

Infected LV thrombus presents considerable diagnostic and therapeutic challenges in clinical practice. Patients with advanced heart failure, particularly those with low ejection fraction, present increased thromboembolic risks due to impaired myocardial contractility and intracardiac stasis.^1,2^ Infection of intracardiac thrombi significantly complicates management due to ongoing bacterial seeding, persistent bacteraemia, systemic embolism, and increased mortality.^2^

Persistent bloodstream infections, as illustrated in our patient, typically indicate a deep-seated source of infection. *S. anginosus* is known for abscess formation and chronic infections, complicating antibiotic penetration and complete eradication.^4^ Traditional antibiotic regimens often fail to eradicate infection due to limited antibiotic penetration within thrombi, necessitating prolonged targeted therapy.

Our case highlights the critical role of multimodal imaging in achieving an accurate diagnosis. TTE is generally the initial modality; however, limitations in sensitivity for thrombi detection, particularly when differentiating valvular and subtle intracardiac lesions, necessitate additional imaging techniques.^1,3^ TOE provides enhanced spatial resolution, particularly for smaller thrombi or subtle endocardial lesions, significantly improving diagnostic accuracy by distinguishing thrombus from valvular vegetations.^5^ Nonetheless, echocardiography alone cannot reliably differentiate infected from sterile thrombi.

PET imaging has emerged as an essential modality for identifying and confirming infected thrombi, characterized by increased FDG uptake in areas of active infection and inflammation.^6^ In our case, PET/CT demonstrated a ring-like configuration with peripheral FDG avidity and central photopenia, consistent with an infected thrombus. This imaging feature likely correlates with inflammatory infiltration at the thrombus periphery and a metabolically inert avascular core, findings not visualizable on echocardiography alone. PET imaging has the unique ability to visualize inflammatory processes at a molecular level, providing critical diagnostic information not accessible via echocardiography alone.^7,8^ While studies evaluating PET sensitivity in infected thrombi are lacking, infective endocarditis, often considered a similar intracardiac infection, has shown variable PET sensitivity depending on the subtype. In a meta-analysis by Wang *et al*., PET demonstrated an overall sensitivity of 74%, but only 31% for native valve endocarditis.^9^ This variability underscores the importance of clinical correlation and highlights the emerging but evolving role of PET in deep-seated cardiac infections. In our case, PET imaging was pivotal in confirming recurrent infected LV thrombus and directly influenced therapeutic decision-making.

Therapeutic anticoagulation represents another area of complexity. Infected thrombi pose dual risks: embolic phenomena requiring anticoagulation and risk of haemorrhagic complications, especially in patients with previous cerebrovascular events.^1^ This patient was previously on apixaban; however, given the development of thrombus despite therapy, treatment failure was suspected. Warfarin was therefore selected for its reversibility and well-established efficacy in left ventricular thrombus.^1^ Bridging with low-molecular-weight heparin was used to reduce the risk of embolization during periods of subtherapeutic anticoagulation.^1,2^

Antimicrobial management of infected intracardiac thrombi is particularly challenging due to the limited penetration of antibiotics into the thrombus core, where organisms may be sequestered within fibrin and biofilm-like structures, analogous to the pathophysiology of infective endocarditis vegetations.^3,10^ In such environments, bacteria are shielded from immune surveillance and antibiotic activity, necessitating extended courses of therapy.^10^ Surgical intervention was not pursued in this case due to the patient’s frailty, multiple comorbidities, and the absence of valvular involvement, making medical management the safer and more appropriate option. Management principles were extrapolated from infective endocarditis, where streptococcal infections, including those caused by the *S. anginosus* group, are recognized as relatively rare but often require prolonged beta-lactam therapy; short-course regimens are not advised.^3^  ^,11^ Accordingly, we implemented a 6 week course of intravenous benzylpenicillin, followed by a 6 month course of oral amoxicillin, tailored to individual sensitivities and guided by input from infectious diseases. The treatment duration was determined by clinical response and aligned with published approaches to deep-seated cardiac infections, where sustained bactericidal therapy is essential to ensure intralesional activity, achieve eradication, and minimize relapse risk.^3,11^

### Conclusion

In summary, we report a complex case of recurrent *S. anginosus* bacteraemia secondary to an infected LV thrombus in a patient with ischaemic cardiomyopathy. To our knowledge, this is among the few reported cases of infected LV thrombus confirmed by PET imaging. This case highlights the diagnostic and therapeutic challenges of managing infected intracardiac thrombi, especially in patients with multiple comorbidities and a history of thromboembolic complications.

Given the nature of thrombotic tissue and the limited antibiotic penetration it affords, management necessitated prolonged intravenous and oral antimicrobial therapy in conjunction with therapeutic anticoagulation. We underscore the importance of early clinical suspicion, the use of comprehensive multimodal imaging, and collaborative decision-making among cardiology, infectious diseases, and imaging specialists. Further research is needed to establish more precise diagnostic criteria, optimal treatment durations, and long-term outcomes in patients with infected LV thrombi.

Future considerations may include studies assessing antibiotic penetration into intracardiac thrombi, the role of adjunctive therapies such as thrombolytics or surgical excision in select patients, and more precise PET imaging criteria for diagnosing thrombus infection. Additionally, the development of specific clinical guidelines for managing infected intracardiac thrombi would support clinicians facing these diagnostically and therapeutically complex scenarios.

## Lead author biography


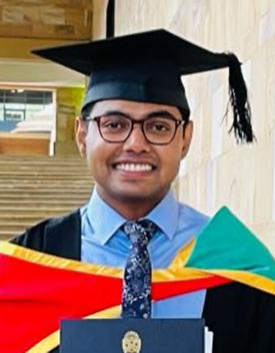


Dr Nithin George is a Basic Physician Trainee based in Adelaide, South Australia, with a strong clinical interest in cardiology, particularly interventional cardiology, heart failure, and multimodality cardiac imaging. He holds a Master of Medicine (Internal Medicine) with a focus on cardiology and clinical epidemiology. Dr George is actively involved in research and medical education for junior doctors and aims to pursue advanced training in cardiology, integrating clinical expertise, evidence-based practice, and innovation to improve cardiovascular outcomes.

## Data Availability

Data from this case report are available on reasonable request. Please contact the corresponding author.

## References

[1] Levine GN, McEvoy JW, Fang JC, Ibeh C, McCarthy CP, Misra A, et al Management of patients at risk for and with left ventricular thrombus: a scientific statement from the American Heart Association. Circulation 2022;146:00–00. 10.1161/CIR.0000000000001092 36106537

[2] McCarthy CP, Murphy S, Venkateswaran RV, Singh A, Chang LL, Joice MG, et al Left ventricular thrombus: contemporary etiologies, treatment strategies, and outcomes. J Am Coll Cardiol 2019;73:2007–2009. 30846340 10.1016/j.jacc.2019.01.031

[3] Delgado V, Ajmone Marsan N, de Waha S, Bonaros N, Brida M, Burri H, et al 2023 ESC guidelines for the management of endocarditis: developed by the task force on the management of endocarditis of the European Society of Cardiology (ESC). Eur Heart J 2023;44:3948–4042. 37622656 10.1093/eurheartj/ehad193

[4] Pilarczyk-Zurek M, Sitkiewicz I, Koziel J. The clinical view on Streptococcus anginosus group – opportunistic pathogens coming out of hiding. Pathogens 2022;11:847. 35898914 10.3389/fmicb.2022.956677 PMC9309248

[5] Habib G, Badano L, Tribouilloy C, Vilacosta I, Zamorano JL; on behalf of the European Association of Echocardiography. Recommendations for the practice of echocardiography in infective endocarditis. Eur J Echocardiogr 2010;11:202–219. 20223755 10.1093/ejechocard/jeq004

[6] Boczar KE, Lau L, Hejji N, Wiefels C. Infective endocarditis: the role of PET imaging in diagnosis and management. J Med Imaging Radiat Sci 2024;55:S17–S25. 38307769 10.1016/j.jmir.2023.12.012

[7] Balmforth C, Whittington B, Tzolos E, Bing R, Williams MC, Clark L, et al Translational molecular imaging: thrombosis imaging with positron emission tomography. J Nucl Cardiol 2024;31:101848. 10.1016/j.nuclcard.2024.101848 38499227

[8] Kenzaka T, Shimoshikiryo M, Kitao A, Kario K, Hashimoto M. Positron emission tomography scan can be a reassuring tool to treat difficult cases of infective endocarditis. J Nucl Cardiol 2011;18:741–743. 21519975 10.1007/s12350-011-9376-x PMC3143318

[9] Wang TKM, Sánchez-Nadales A, Igbinomwanhia E, Cremer P, Griffin B, Xu B. Diagnosis of infective endocarditis by subtype using 18F-fluorodeoxyglucose positron emission tomography/computed tomography: a contemporary meta-analysis. Circ Cardiovasc Imaging 2020;13:e010600. 32507019 10.1161/CIRCIMAGING.120.010600

[10] Murillo H, Restrepo CS, Marmol-Velez JA, Vargas D, Ocazionez D, Martinez-Jimenez S, et al Infectious diseases of the heart: pathophysiology, clinical and imaging overview. RadioGraphics 2016;36:963–983. 27399236 10.1148/rg.2016150225

[11] Baddour LM, Wilson WR, Bayer AS, Fowler VG, Tleyjeh IM, Rybak MJ, et al Infective endocarditis in adults: diagnosis, antimicrobial therapy, and management of complications. Circulation 2015;132:1435–1486. 26373316 10.1161/CIR.0000000000000296

