# Correction to: Infected left ventricular thrombus confirmed by FDG PET/CT presenting as persistent *Streptococcus anginosus* bacteraemia in ischaemic cardiomyopathy: a case report

**DOI:** 10.1093/ehjcr/ytaf625

**Published:** 2025-12-08

This is a correction to: Nithin George, Infected left ventricular thrombus confirmed by FDG PET/CT presenting as persistent *Streptococcus anginosus* bacteraemia in ischaemic cardiomyopathy: a case report, *European Heart Journal - Case Reports*, Volume 9, Issue 11, November 2025, https://doi.org/10.1093/ehjcr/ytaf579

The following change has been made to the originally published manuscript.

A header “**Conclusion**” has been inserted into the paper to indicate where that section begins.

